# Complete Chloroplast Genomes of *Ampelopsis humulifolia* and *Ampelopsis japonica*: Molecular Structure, Comparative Analysis, and Phylogenetic Analysis

**DOI:** 10.3390/plants8100410

**Published:** 2019-10-14

**Authors:** Xiaolei Yu, Wei Tan, Huanyu Zhang, Han Gao, Wenxiu Wang, Xiaoxuan Tian

**Affiliations:** Tianjin State Key Laboratory of Modern Chinese Medicine, Tianjin University of Traditional Chinese Medicine, Tianjin 300193, China; yuxiaolei127@163.com (X.Y.); tanwei0817@163.com (W.T.); zhanghychn@163.com (H.Z.); kafeihb@sina.com (H.G.); wxx1272071351@163.com (W.W.)

**Keywords:** *Ampelopsis humulifolia*, *Ampelopsis japonica*, chloroplast genome, phylogenetic analysis, molecular structure

## Abstract

*Ampelopsis humulifolia* (*A. humulifolia*) and *Ampelopsis japonica* (*A. japonica*), which belong to the family Vitaceae, are valuably used as medicinal plants. The chloroplast (cp) genomes have been recognized as a convincing data for marker selection and phylogenetic studies. Therefore, in this study we reported the complete cp genome sequences of two *Ampelopsis* species. Results showed that the cp genomes of *A. humulifolia* and *A. japonica* were 161,724 and 161,430 bp in length, respectively, with 37.3% guanine-cytosine (GC) content. A total of 114 unique genes were identified in each cp genome, comprising 80 protein-coding genes, 30 tRNA genes, and 4 rRNA genes. We determined 95 and 99 small sequence repeats (SSRs) in *A. humulifolia* and *A. japonica*, respectively. The location and distribution of long repeats in the two cp genomes were identified. A highly divergent region of *psbZ* (Photosystem II reaction center protein Z) -*trnG* (tRNA-Glycine) was found and could be treated as a potential marker for Vitaceae, and then the corresponding primers were designed. Additionally, phylogenetic analysis showed that *Vitis* was closer to *Tetrastigma* than *Ampelopsis*. In general, this study provides valuable genetic resources for DNA barcoding marker identification and phylogenetic analyses of *Ampelopsis*.

## 1. Introduction

The *Ampelopsis* comprises approximately thirty species that are distributed in Asia, North America, and Central America, mainly distributed in hilly land, bush or meadow at an approximate altitude of 100–1100 m above sea level [1]. *A. humulifolia* and *A. japonica* have high medicinal value, and the whole body or the dried roots of two plants are frequently used to treat inflammation in China [1,2]. Moreover, *A. japonica* possesses antimicrobial activity, antitumor activity, immunomodulatory, and excitatory function [3,4,5]. Thanks to the development of next generation sequencing technologies, the attention of plant molecular research has been increasing in recent years [6,7]. Whereas, the literature report of the chloroplast (cp) genome from the *Ampelopsis* is extremely scarce. Up to now, only one cp genome from *Ampelopsis* has been reported [8], hindering molecular research on *Ampelopsis*.

The cp genome encodes a group of proteins involved in photosynthesis and other biochemical pathways that play an important role in plant growth, development and evolution [9]. Substitution rates in the nucleotides of cp genome are much lower than in nuclear DNA, low levels of recombination, and primarily uniparental inheritance make cp genome a useful tool for DNA barcoding and valuable source of genetic data for phylogenetic analyses [10,11]. In recent years, the complete cp genome sequences have been widely used for molecular identification, phylogenetic studies, genetic engineering, and increasing phylogenetic resolution at low taxonomic levels [12,13]. Although universal markers such as *matK* (megakaryocyte-associated tyrosine kinase), *rbcL* (ribulose bisphosphate carboxylase large subunit), *psbA* (Photosystem II reaction center protein A) -*trnH* (tRNA-Histidine), and *ycf1* (yeast cadmium factor 1) are frequently used for the identification of plants [14,15], the efficiency of species identification varies from family to family [16,17]. In China, the dried roots of *Ampelopsis* species are often used to treat inflammation, whereas the dried roots of *Ampelopsis* species lose their morphological features, making it hard for species identification. Therefore, it is necessary to find a potential marker which is suitable for Vitaceae.

Here, we first reported the cp genomes of two *Ampelopsis* (*A. humulifolia* and *A. japonica*) of the family Vitaceae. We also detected the long repeats and small sequence repeats (SSRs) in the genomes, including distribution patterns, distribution locations, and repeat types. Comparative sequences analyses and highly divergent regions were analyzed for three *Ampelopsis* species and six Vitaceae species. Furthermore, we reconstructed a phylogenetic tree based on 80 protein-coding genes to evaluate the phylogenetic relationships within Vitaceae. Our aims are: (1) to characterize the cp genomes of *A. humulifolia* and *A. japonica*; (2) to provide an insight into the evolutionary analysis of Vitaceae; (3) to identify and screen molecular markers suitable for species identification; (4) to provide a deep-level phylogenetic analysis of *Ampelopsis*.

## 2. Results and Discussion

### 2.1. Characteristics of the Chloroplast Genomes of A. humulifolia and A. japonica

**Figure 1:**
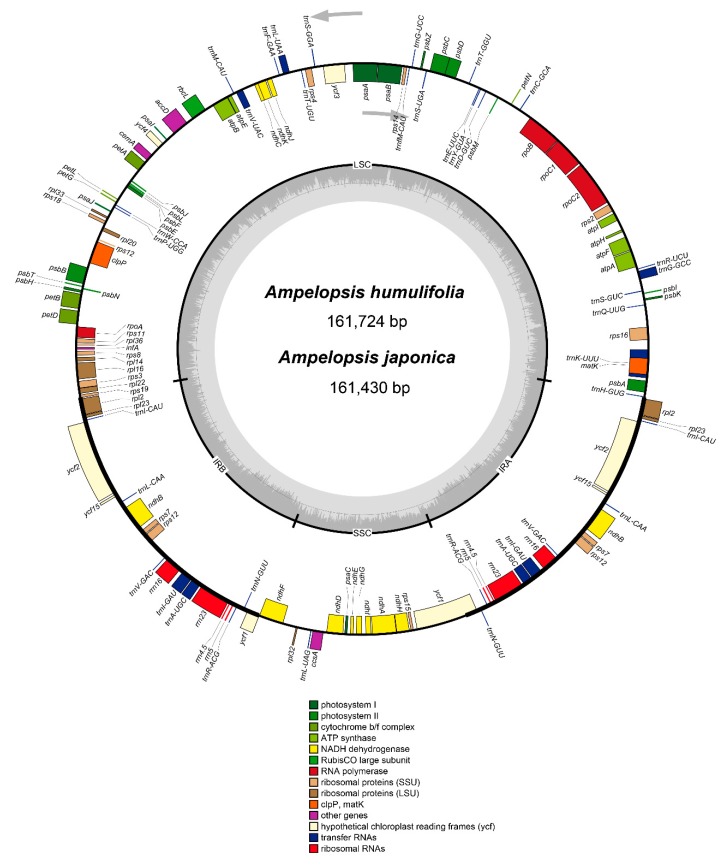
Gene map of the complete chloroplast (cp) genomes of *A. humulifolia* and *A. japonica*. Genes inside the circle are transcribed clockwise, whereas those on the outside are transcribed counter-clockwise. Genes belonging to different functional groups are color-coded. Large single copy (LSC), small single copy (SSC), IRa, and IRb are indicated. The darker grey in the inner circle represents the guanine-cytosine (GC) content, while the lighter grey represents the AT content.

The cp genomes of *A. humulifolia* and *A. japonica* were 161,724 and 161,430 bp in length, respectively. Both species exhibited a typical quadripartite structure built with four regions: large single copy (LSC), small single copy (SSC), and two inverted repeats (IRa and IRb) (Figure 1): Two single-copy regions (LSC 89,650 and 89,626 bp, SSC 19,032 and 18,977 bp in *A. humulifolia* and *A. japonica*, respectively) separated by a pair of IRs (26,521 and 26,413 bp in *A. humulifolia* and *A. japonica*, respectively) (Figure 1 and Table 1). The guanine-cytosine (GC) content of the two cp genomes was 37.3 %, and the IR regions had the highest GC content (42.9% in both species). The GC content of LSC (35.2% in both species) and SSC (31.8% and 31.9% in *A. humulifolia* and *A. japonica*, respectively) regions was lower than the IR regions (42.9% in both species) (Table 1). The cp genome features of *A. humulifolia* and *A. japonica* were consistent with another *Ampelopsis* species in terms of genomic structure [8].

A total of 114 unique genes are present in the two cp genomes, including 80 protein-coding genes, 30 tRNA genes, and 4 rRNA genes (Table 1). Among these genes, eighteen genes were duplicated in the IR regions, including seven protein-coding genes, seven tRNA, and four rRNA genes (Table 2). There were eighteen genes with introns, and sixteen of which contained one intron (*atpF, ndhA, ndhB, petB, petD, rpoC1, rps12, rps16, rpl16, rpl2, trnA-UGC, trnG-UCC, trnI-GAU, trnK-UUU, trnL-UAA, trnV-UAC*), while two genes (*clpP and ycf3*) contained two introns (Table 2).

### 2.2. Codon Usage Analysis

**Figure 2:**
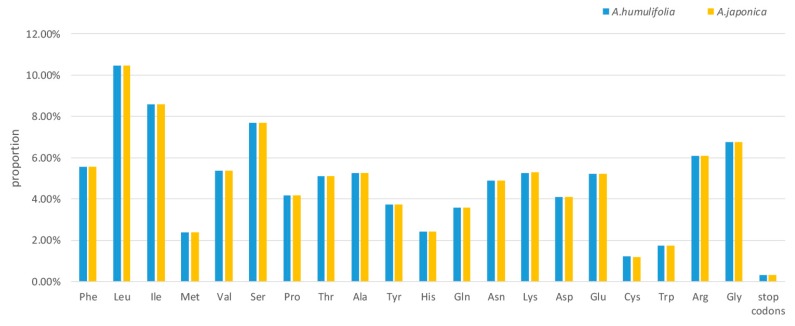
Amino acid proportion in *A. humulifolia* and *A. japonica* protein-coding sequences.

The analysis of codon usage is valuable in shaping chloroplast genome evolution [18]. The shared protein-coding genes contained 26,998 and 26,990 codons in the cp genomes of *A. humulifolia* and *A. japonica*, respectively (Table S1). Among these codons, 2824 (10.46%) in *A. humulifolia* and 2821 (10.45%) in *A. japonica* encoded leucine, whereas only 326 (1.21%) in *A. humulifolia* and 325 (1.20%) in *A. japonica* codons encoded cysteine (Figure 2 and Table S1). The AT content for the first, second, and third codon positions, respectively, was 54.44%, 61.85%, and 69.95% in *A. humulifolia* and 54.43%, 61.95%, and 69.97% in *A. japonica*. Additionally, the two cp genomes shared identical features: (1) All types of preferred synonymous codons (Relative synonymous codon usage, RSCU >1.00) ended with A or U, except UGG. (2) The codons ATG and TGG, encoding methionine and tryptophan, respectively, exhibited no bias (RSCU = 1.00) (Table S1). Our study revealed that the bias presented higher AT content in the third position of the codon, which was consistent with other land plants’ cp genomes [19].

### 2.3. Analysis of SSRs and Long Repeats

**Figure 3:**
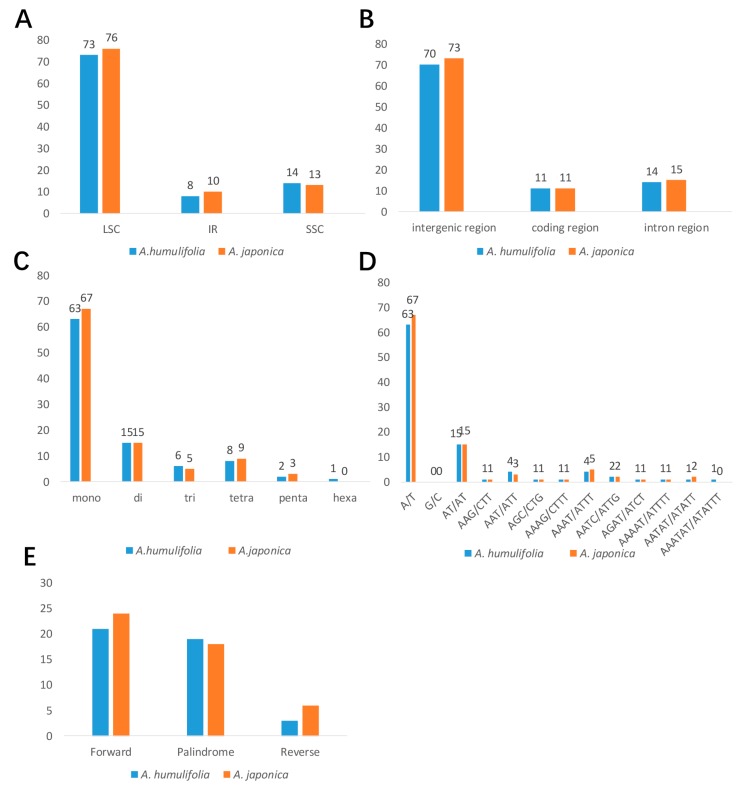
Analyses of repeats in the cp genomes of *A. humulifolia* and *A. japonica*. (**A**) Distribution of SSRs in the LSC, IR, and SSC. (**B**) Distribution of SSRs in the intergenic region, coding region, and intron region. (**C**) Number of different types of SSRs. (**D**) Number of different repeat units of SSRs. (**E**) Number of different types of long repeats.

SSR has been described as a major tool for investigating genomic polymorphism across species and for population genetic studies within species in plant molecular studies [20,21]. A total of 95 and 99 SSRs (The minimum repeat numbers of SSRs for mono-, di- tri- tetra-, penta-, and hexanucleotides are 10, 5, 4, 3, 3, and 3, respectively) were found in the cp genomes of *A. humulifolia* and *A. japonica*, respectively (Figure 3A). Among those repeats, the content of mononucleotide SSRs was the richest (63 and 67 in *A. humulifolia* and *A. japonica*, respectively), and all of these mononucleotide SSRs were A or T. Moreover, most SSRs were located in the single copy region, especially in the LSC region (73 and 76 in *A. humulifolia* and *A. japonica*, respectively). In the genomic structure of two *Ampelopsis* species, the intergenic region had the most abundant SSRs, whereas the coding region had the least SSRs (Figure 2B). Additionally, we detected tandem repeats in the cp genomes of two *Ampelopsis* species. The tandem repeats were mainly distributed in the intergenic region (Table S2). Moreover, we identified 43 (21 forward, 19 palindrome, and 3 reverse) and 48 (24 forward, 18 palindrome, and 6 reverse) long repeats (repeat size ≥ 30 bp) in *A. humulifolia* and *A. japonica*, respectively (Figure 3E and Table S3). Approximately 50% of the long repeats in *A. humulifolia* and 45% of the long repeats in *A. japonica* were completely located within genes. Among these genes, *ycf2* had the largest number of long repeats.

### 2.4. Comparative Genome Analysis

**Figure 4:**
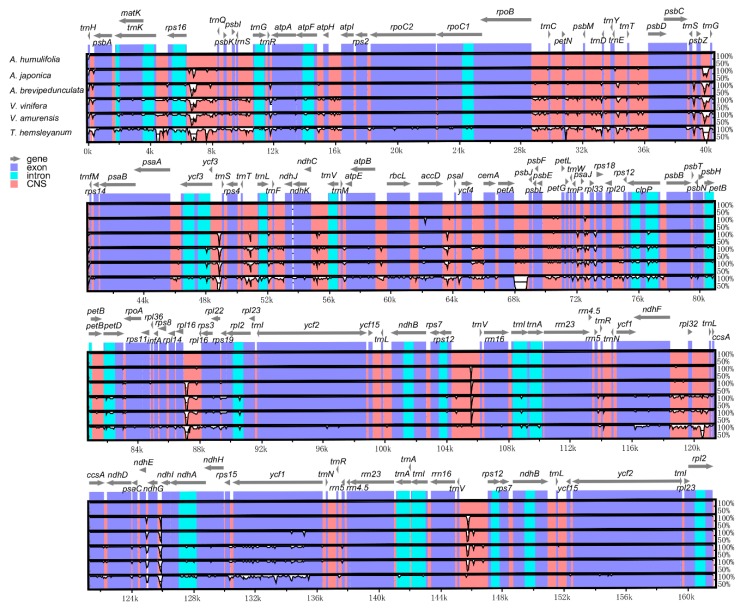
Comparison of six chloroplast genomes, with *A. humulifolia* as a reference using mVISTA alignment program. Grey arrows above the alignment present the orientation of genes. Violet bars indicate exons, cyan bars indicate introns, and salmon bars indicate non-coding sequences (CNS). The y-axis indicates the identity percentage ranging from 50 to 100%.

To make comparative genome analysis, the complete cp genome sequences of *A. humulifolia* and *A. japonica* were compared with *A. brevipedunculata*, *Vitis vinifera* (*V. vinifera*), *Vitis amurensis* (*V. amurensis*), and *Tetrastigma hemsleyanum* (*T. hemsleyanum*) using the mVISTA program (Figure 4). As expected, the coding regions were more conserved than the non-coding regions. Furthermore, the highly divergent regions were mainly located in the intergenic regions, such as *rps16-trnQ* and *psbZ-trnG*. The highly divergent coding-regions were *rbcL*, *rpl22*, *accD*, and *ycf1*. Overall, the genomic structure of the six Vitaceae species presented a high degree of synteny and gene order conservation, suggesting the evolutionary conservation of these species at the genome-scale level.

### 2.5. Nucleotide Diversity Analysis

**Figure 5:**
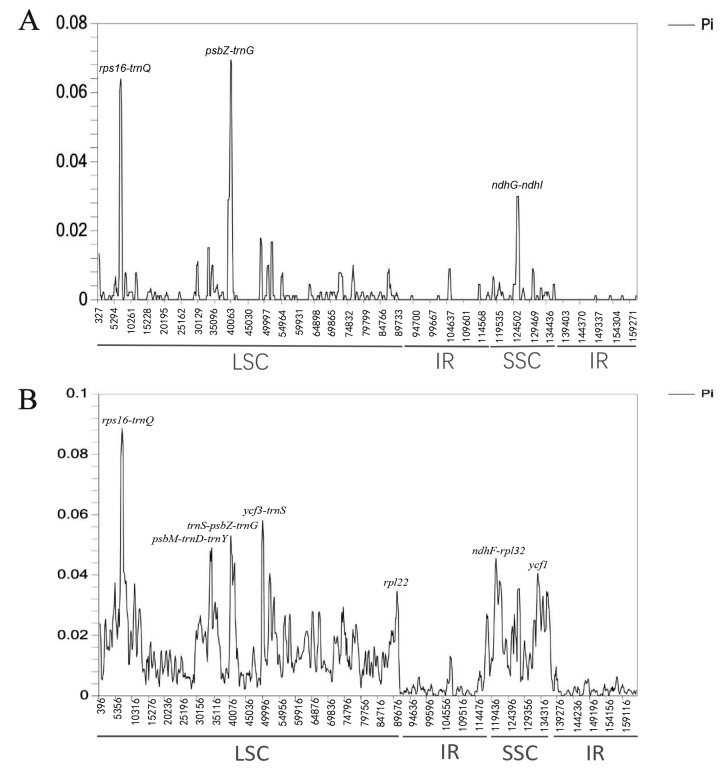
Sliding window analysis of the cp genomes. (**A**) Comparison of the Pi among cp genomes of *A. humulifolia*, *A. japonica*, and *A. brevipedunculata*. (**B**) Comparison of the Pi among six Vitaceae species’ cp genomes.

To investigate the sequence divergence of cp genomes, we calculated the nucleotide variability (Pi) value among three *Ampelopsis* species and six Vitaceae species (Figure 5). The results showed that the variability of the IR region was less than that of the LSC and SSC regions in the six Vitaceae species. Three highly divergent regions were found among three *Ampelopsis* species, which were *rps16-trnQ*, *psbZ-trnG*, and *ndhG-ndhI*. Among them, the most highly divergent region was *psbZ-trnG*, with the Pi value of 0.069. Furthermore, there were eight highly divergent regions detected among the six Vitaceae species, including six intergenic regions as *rps16-trnQ*, *psbM-trnD-trnY*, *trnS-psbZ*, *ycf3-trnS, ndhF-rpl32*, and two coding regions as *rpl22* and *ycf1*. The *rps16-trnQ* region was the highest divergent region with the Pi value of 0.088.

### 2.6. Marker Comparison

We selected markers based on the highly variable regions of the coding-region, intron, intergenic region, and designed the corresponding primers for species identification (Table S4). We extracted the universal marker regions of *matK*, *rbcL*, *psbA-trnH*, and *ycf1* as described in previous studies [14,22]. All designed primers were evaluated with high scores using Oligo 7 software. The result indicated that the *clpP* intron, the *psbZ-trnG*, and the *rps16-trnQ* showed higher mean K2P values than recommended plant markers among six Vitaceae species, and the *psbZ-trnG* and *rps16-trnQ* showed higher mean K2P values than recommended plant markers among three *Ampelopsis* species, indicating the high divergence of *psbZ-trnG* and *rps16-trnQ* in the family and genus levels. The *psbZ-trnG* and *rps16-trnQ* had lower mean length of the marker region than recommended plant markers of *matK* and *rbcL*. The average similarity of primers for *psbZ-trnG* is higher than that of *matK* and *rbcL*. The result showed that *psbZ-trnG* was the most suitable potential marker due to its features of high variation, conserved primers, and short length.

### 2.7. IR Expansion and Contraction

**Figure 6:**
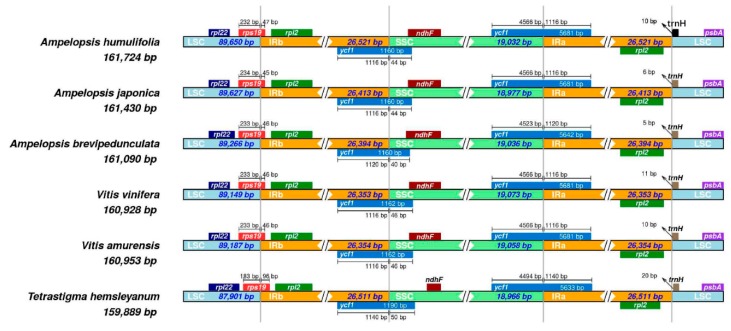
Comparison of the borders of LSC, SSC, and IR regions among six chloroplast genomes.

The expansion and contraction of the border regions have been thought to be the evolutionary events and be responsible for changes in chloroplast genome size [23,24]. We compared the border regions and their adjacent genes among six Vitaceae species (Figure 6). The rps19 gene was located at the junction of LSC/IRb, extending 45–47 bp into the IRb region. The *ycf1* gene was located at the junction of the IRb/SSC and extended 44–46 bp into the SSC region. The *trnH* gene entirely was located in the LSC, 5–11 bp away from the IRa/LSC border. Overall, the genomic structure of the six Vitaceae species was consistent, while the length difference of the four regions (LSC, SSC, IRa, and IRb) resulted in the size of the six genomes ranging from 159, 889 to 161, 724 bp.

### 2.8. dN/dS Ratio and Kimura 2-Parameter (K2P) Genetic Distance

**Figure 7:**
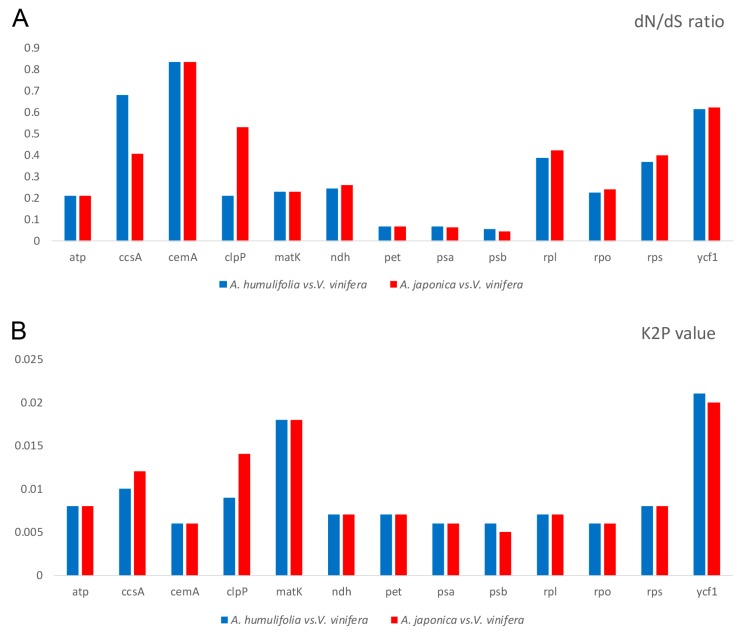
Evolutionary dynamics of gene groups and some genes of *A. humulifolia* vs. *V. vinifera* and *A. japonica* vs. *V. vinifera*. (**A**) dN/dS ratio for gene groups and some genes; (**B**) The Kimura 2-parameter (K2P) value for gene groups and some genes.

The synonymous (dS) and nonsynonymous (dN) substitution ratios are valuable in understanding the dynamics of molecular evolution [25]. The ratios of dN/dS > 1, < 1 and = 1 indicate the positive selection, negative selection, and neutral selection, respectively. Higher dN / dS ratios indicate a faster evolution of genes. The dN/dS ratios were calculated based on gene groups and some genes in *A. humulifolia* vs. *V. vinifera* and *A. japonica* vs. *V. vinifera*, respectively (Figure 7). Both calculations used *V. vinifera* as the outgroup. The dN/dS ratios of all protein-coding genes were less than 1, indicating that these genes were under negative selection. As for the consistent results of two comparisons, the photosynthetic apparatus genes (*pet*, *psa*, *psb*) had significantly low dN/dS ratios, the *ycf1* gene showed high dN/dS ratios, the RNA processing gene (*matK)*, ATP synthase genes (*atp*), NAPH dehydrogenase gene (*ndh*), and RNA polymerase gene (*rpo*) showed moderate dN/dS ratios. As for *A. humulifolia* vs. *V. vinifera*, the *ccsA* for cytochrome c synthesis and *cemA* for carbon metabolism had high dN/dS ratios. As for *A. japonica* vs. *V. vinifera*, the *cemA* and *clpP* for proteolysis showed high dN/dS ratios. The high dN/dS ratios of *ycf1* indicated high variability of *ycf1*, and the high variability of *ycf1* encoded proteins was commonly found in other land plants [14,26]. The comparisons of *A. humulifolia* vs. *V. vinifera* and *A. japonica* vs. *V. vinifera* possessed identical features: (1) The photosynthetic apparatus genes (*pet*, *psa*, *psb*) showed strongly negative selection during evolution; (2) the RNA processing gene (*matK*), ATP synthase gene (*atp*), NAPH dehydrogenase gene (*ndh*), and RNA polymerase gene (*rpo*) showed a moderate rate of evolution; (3) the *ycf1* gene showed a high rate of evolution. Based on Kimura 2-parameter (K2P) model, we calculated the interspecific genetic distance of *A. humulifolia* vs. *V. vinifera* and *A. japonica* vs. *V. vinifera*, respectively (Figure 7). We found that the photosynthetic apparatus genes (*pet, psa, psb*) showed low K2P values, in contrary, the *ycf1* showed the highest K2P values. The results were similar to the analysis of dN/dS.

### 2.9. Phylogenomic Analysis

**Figure 8:**
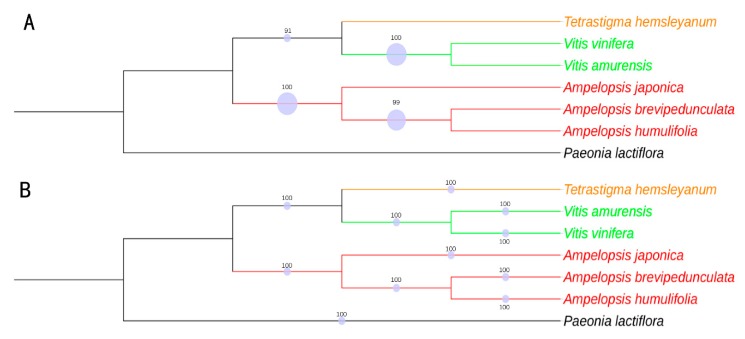
Phylogenetic trees of six Vitaceae species based on 80 protein-coding genes, the *Paeonia lactiflora* was used as an outgroup. (**A**) Maximum likelihood (ML) phylogenetic tree constructed with 80 protein-coding genes; (**B**) bayesian inference (BI) phylogenetic tree constructed with 80 protein-coding genes.

With the development of sequencing technologies, more and more cp genome sequences were used to reconstruct plant phylogenies [27,28]. Considering that the outgroup determines the polarization of character states, multiple outgroups may turn singletons into informative sites, which might change the topology of a tree [29]. The *Paeonia lactiflora* was set as an outgroup, due to its close relationship with Vitaceae [30]. In this study, 80 shared protein-coding genes of seven species (six Vitaceae species and one outgroup) were used to reconstruct phylogenetic trees using the Maximum likelihood (ML) and Bayesian inference (BI) methods (Figure 8). All nodes in the ML tree and BI tree had high bootstrap support values. As for *Ampelopsis*, the ML tree indicated that *A. humulifolia* and *A. brevipedunculata* were clustered into one clade with a bootstrap value of 99%, and *A. japonica* formed an independent clade. The result indicated the affinity relationship between *A. humulifolia* and *A. brevipedunculata*, which was coincident with a previous study using four plastid regions (*trnL-F*, *rps16*, *psbA-trnH*, and *atpB-rbcL*) [31]. As for Vitaceae, Tetrastigma and Vitis formed a clade with 91% bootstrap value in the ML tree. The BI tree showed consistent results with the ML tree with support values of 100%. Our results were consistent with previous studies that *Vitis* was closer to *Tetrastigma* than *Ampelopsis* [31,32,33,34]. Overall, our research provided a useful resource for molecular study within the family Vitaceae.

## 3. Materials and Methods

### 3.1. DNA Sequencing and Genome Assembly

The *A. humulifolia* and *A. japonica* plant samples were collected from Yao Mountain of Tianjin University of Traditional Chinese Medicine, Tianjin, China, and were identified by Prof. Tianxiang Li from School of Chinese Materia Medica, Tianjin University of Traditional Chinese Medicine. The voucher species were deposited in Tianjin State Key Laboratory of Modern Chinese Medicine, Tianjin University of Traditional Chinese Medicine. Total genomic DNA was extracted using Extract Genomic DNA Kit (Sangon Biotech Co., Ltd., Shanghai, China) and was used to construct cp DNA library using small fragments with an average insert size of 350 bp according to the manufacturer’s instructions (Novogene, Nanjing, China). The libraries were sequenced with 2 × 150 bp on the Illumina Hiseq instrument (Novogene, Nanjing, China). The resulting raw data were assessed with FastQC. Trimmed paired-end reads were assembled using CLC Genome Assembler (v11.0.1, CLC Inc, Aarhus, Denmark) with default parameters. Cp genome sequence contigs were selected from the initial assembly by performing a BLAST search (http://blast.ncbi.nlm.nih.gov/) using the *Ampelopsis brevipedunculata* (A. *brevipedunculata*) chloroplast genome sequence as a reference (GenBank accession KT831767). Based on their similarity, CP-like reads were extracted and assembled to complete cp genomes by mapping to reference, then the generated circular genomes were remapped to raw data to verify the assembly using CLC Genome Assembler. The sequencing results were listed in Table S5.

### 3.2. Genome Annotation

The initial cp genome annotations were performed using DOGMA (http://dogma.ccbb.utex-as.edu/) [35] and confirmed with CPGAVAS (http://www.herbalgenomics.org/cpgavas) [36]. The tRNA genes were identified using tRNAscanSE (http://lowelab.ucsc.edu/tRNAscan-SE/) [37] with default settings. Then, all of the annotations were manually adjusted against the reference (KT831767, KX499471, NC_007957). Finally, the circular chloroplast genome maps were depicted using OGDRAW (http://ogdraw.mpimp-golm.mpg.de/) [38]. The NCBI accession numbers of *A. japonica* and *A. humulifolia* are MK547541 and MK547542, respectively.

### 3.3. Genome Structure Analysis

Codon usage, RSCU and GC content were determined using MEGA7 [39]. The software MISA [40] was used to detect SSRs, using the following parameters: 10, 5, 4, 3, 3, and 3 for mono-, di-, tri- tetra-, penta-, and hexanucleotides, respectively. The size and location of repeat sequences, including forward, palindromic, reverse, and complement repeats, were determined by REPuter (http://bibiserv.tech--fak.uni-bielefeld.de/reputer/) [41], with a minimal repeat size of 30 bp, and a Hamming distance of 3. Tandem Repeats Finder (http://tandem.bu.edu/trf/trf.submit.options.html) [42] was used to find tandem repeats with default settings.

### 3.4. Genome Comparison

To make comparative analysis in the cp genome level between *A. humulifolia*, *A. japonica*, *A. brevipedunculata*, *V. vinifera*, *V.amurensis*, and *T. hemsleyanum*, four cp genomes were downloaded from NCBI (Table S6), and mVISTA program [43] was used with a LAGAN mode. The nucleotide variability (Pi) among cp genomes was performed using DnaSP v6.11.01 software [44], with a window length of 600 bp and a step size of 200 bp. The primers were designed and evaluated by Primer 5.0 software (Premier Biosoft International, PaloAlto, CA) and Oligo 7 software (Molecular Biology Insights, DBA), respectively. The online tool IRscope (https://irscope.shi-nyapps.io/irapp/) [45] was used to visualize the genes on the boundaries of LSC, SSC, and IRs according to their annotations.

### 3.5. Analysis of dN/dS Ratio and K2P Genetic Distance

The same functions of the genes were grouped following previous studies to analyze nonsynonymous substitution rate (dN/dS) [46,47,48]. Analyses were carried based on the same function genes (*atp, ndh, pet, psa, psb, rpl, rpo*, and *rps*) and singular genes (*ccsA, clpP, cemA, matK*). The PAML v4.9 package [49] was used to evaluate dN/dS ratio, and *V. vinifera* was set as an outgroup. MEGA7 [39] was used to calculate the interspecific genetic distance based on the K2P model, and *V. vinifera* was used as a reference.

### 3.6. Phylogenetic Analysis

In this study, seven species (six Vitaceae species and one outgroup) were selected to construct a phylogenetic tree, five of which were downloaded from NCBI database (Table S6). The 80 shared protein-coding genes of seven species were extracted, aligned separately, and recombined to construct a matrix using PhyloSuite_v1.1.15 [50]. Then, the matrix was used to construct the phylogenetic tree, and *Paeonia lactiflora* was set as the outgroup. RA × ML version 8.2.4 [51] was used to perform ML analysis with 1000 bootstrap replicates and GTR + G substitution model. The BI analysis was implemented with MrBayes 3.2.2 [52], and the best-fitting model (GTR + G) was determined with jModeltest [53] based on the Akaike Information Criterion (AIC). The final trees were visualized in the Interactive Tree Of Life (https://itol.embl.de) [54].

## 4. Conclusions

In this study, we characterized the complete chloroplast genomes of two *Ampelopsis* (*A. humulifolia* and *A. japonica*) of the family Vitaceae. The codon usage, GC content, microsatellites, and repeat sequences were determined in the two cp genomes. The comparison of six Vitaceae cp genome sequences revealed the evolutionary conservation of these species at the genome-scale level. The expansion and contraction of the border regions resulted in difference of genome size between six Vitaceae species. The analyses of *dN/dS ratio* showed that all selected genes were under negative selection during evolution. The phylogenetic analyses showed that *A. humulifolia* was a sister-branch to *A. brevipedunculata*, and *Vitis* was a sister-branch to *Tetrastigma*. The highly divergent region of *psbZ-trnG* was found and can be treated as a potential marker for Vitaceae species, and the corresponding primers were designed. Overall, this study will provide useful genetic information of *Ampelopsis* and contribute to further investigation of Vitaceae.

## Figures and Tables

**Table 1:** Features of the chloroplast genomes of *A. humulifolia* and *A. japonica*

Species	*A. humulifolia*	*A. japonica*
Genome size (bp)	161,724	161,430
LSC length (bp)	89,650	89,626
IR length (bp)	26,521	26,413
SSC length (bp)	19,032	18,977
Number of genes	114	114
Protein-coding genes	80	80
tRNA genes	30	30
rRNA genes	4	4
Total GC content (%)	37.3	37.3
LSC	35.2	35.2
IR	42.9	42.9
SSC	31.8	31.9

**Table 2:** Genes present in *A. humulifolia* and *A. japonica* chloroplast genomes.

Category	Gene Name
Photosystem I	*psaA, psaB, psaC, psaI, psaJ*
Photosystem II	*psbA, psbB, psbC, psbD, psbE, psbF, psbH, psbI, psbJ, psbK, psbL, psbM, psbN, psbT, psbZ*
Cytochrome b/f complex	*petA, petB^b^, petD^b^, petG, petL, petN*
ATP synthase	*atpA, atpB, atpE, atpF^b^, atpH, atpI*
NADH dehydrogenase	*ndhA^b^, ndhB^a,b^, ndhC, ndhD, ndhE, ndhF, ndhG, ndhH, ndhI, ndhJ, ndhK*
RNA polymerase	*rpoA, rpoB, rpoC1^b^, rpoC2*
Large subunit ribosomal proteins	*rpl2^a^, rpl14, rpl16^b^, rpl20, rpl22, rpl23 ^a^, rpl32, rpl33, rpl36*
Small subunit ribosomal proteins	*rps2, rps3, rps4, rps7^a^, rps8, rps11, rps12^a,c^, rps14, rps15, rps16^b^, rps18, rps19*
Ribosomal RNA genes	*rrn4.5^a^, rrn5^a^, rrn16^a^, rrn23^a^*
Transfer RNA genes	*trnA-UGC^a,b^, trnC-GCA, trnD-GUC, trnE-UUC, trnF-GAA, trnfM-CAU, trnG-GCC, trnG-UCC^b^, trnH-GUG, trnI-CAU^a^, trnI-GAU^a,b^, trnK-UUU^b^, trnL-CAA^a^, trnL-UAA^b^, trnL-UAG, trnM-CAU, trnN-GUU^a^, trnP-UGG, trnQ-UUG, trnR-ACG^a^, trnR-UCU, trnS-GGA, trnS-UGA, trnT-GGU, trnT-UGU, trnV-GAC^a^, trnV-GAU, trnV-UAC^b^, trnW-CCA, trnY-GUA*
Fatty acid synthesis	*accD*
Carbon metabolism	*cemA*
Cytochrome c synthesis	*ccsA*
Proteolysis	*clpP^c^*
Translation initiation factor	*infA*
RNA processing	*matK*
Rubisco	*rbcL*
Proteins of unknown function	*ycf1^a^, ycf2^a^, ycf3^c^, ycf4, ycf15 ^a^*

a—Two gene copies in IRs; b—Genes with one intron; c—Genes with two introns.

## Supplementary Materials

Supplementary materials can be found at https://www.mdpi.com/2223-7747/8/10/410/s1. Table S1: Codon usage; Table S2: Tandem repeats; Table S3: Long repeats; Table S4: Marker comparison; Table S5: Sequencing results; Table S6: NCBI accession number.
