# Supplementary material for: Complete Chloroplast Genomes of Ampelopsis humulifolia and Ampelopsis japonica: Molecular Structure, Comparative Analysis, and Phylogenetic Analysis
Source: Plants (Basel). 2019 Oct 14;8(10):410. doi: 10.3390/plants8100410 (PMC6843361; doi:10.3390/plants8100410)
Supplement: Supplementary file 1 [file plants-08-00410-s001.zip › plants-594329-SI/supplementary materials/Table S4. Marker comparison.docx]

**Table S4.** Comparison of the sequences between universal markers and potential markers

| **Marker** | **Mean K2P value among Six Vitaceae species** | | **Mean K2P value among three *Ampelopsis* species** | | **Mean length of marker region among six Vitaceae species (bp)** | | **Mean similarity of primer among six Vitaceae species (%)** | | | **Mean similarity of primer among three Ampelopsis species (%)** | | **Primer pair** | **Oligo score** | | **Primer source** |  |
| --- | --- | --- | --- | --- | --- | --- | --- | --- | --- | --- | --- | --- | --- | --- | --- | --- |
| *matK*  (CDS)  *rbcL*  (CDS)  *psbA-trnH*  (IGS)  *ycf1*  (CDS)  *accD*  (CDS)  *clpP*  (intron)  *rps16-trnQ*  (IGS)  *psbZ-trnG*  (IGS)  *rpl22*  (CDS) | | | 0.014  0.012  0.032  0.036  0.027  0.045  0.115  0.117  0.030 | | N/A  N/A  0.016  0.003  0.013  0.013  0.279  0.129  N/A | | 892  703  421  879  230  374  595  425  347 | | 93.2  97.5  100  91.5  99.2  100  95.9  99.6  98 | 93.2  97.5  100  92.2  100  100  95.1  99.2  100 | | Forward: CGATCTATTCATTCAATATTTC  Reverse:  TCTAGCACACGAAAGTCGAAGT  Forward:  ATGTCACCACAAACAGAAAC  Reverse:  TCGCATGTACCTGCAGTAGC  Forward:  GTTATGCATGAACGTAATGCTC  Reverse:  CGCGCATGGTGGATTCACAATCC  Forward:  TCTCGACGAAAATCAGATTGTTGTGAAT  Reverse:  ATACATGTCAAAGTGATGGAAAA  Forward:  TTATTCGGCGTCAGGGACAT  Reverse:  AACGATCATAGTGGGGATGA Forward:  GGAACCGTACATGCACCTTT  Reverse:  GTCGATCGGATCTTTACCCGG Forward:  CATTCCTCTAGTTTGGAGCC  Reverse:  TGTGTCACTTGAAGATGCAGA Forward:  GAAAGAATTCGGGGGAGGGG  Reverse:  CAGAGCCGAGCCAGATACTC Forward:  CCAAGTCTGAAACCCAGTGGA  Reverse:  TCGTGGACGTTCCTATCAGGA | | | 458  704  722  609  741  228  707  671  401  819  668  771  810  728  807  714  894  462 | Chen *et al.*  Chen *et al.*  Chen *et al.*  Dong *et al.*    This study  This study  This study  This study  This study |

CDS-coding region; IGS-intergenic region; N/A-region with 100% identity.
