# Supplementary material for: Complete Chloroplast Genomes of Ampelopsis humulifolia and Ampelopsis japonica: Molecular Structure, Comparative Analysis, and Phylogenetic Analysis
Source: Plants (Basel). 2019 Oct 14;8(10):410. doi: 10.3390/plants8100410 (PMC6843361; doi:10.3390/plants8100410)
Supplement: Supplementary file 1 [file plants-08-00410-s001.zip › plants-594329-SI/supplementary materials/Table S5. Sequencing results.docx]

**Table** **S5.** Sequencing results of the chloroplast genomes of *A. humulifolia* and *A. japonica*

| **Species** | ***A. humulifolia*** | ***A. japonica*** |
| --- | --- | --- |
| Total reads  Assembled reads  Average coverage | 35,929,922  1,142,007  1,058 | 42,714,594  1,520,686  1,413 |
